# The outcomes of secondary AML post allogeneic hematopoietic cell transplantation significantly depend on the presence of poor‐risk cytogenetic abnormalities

**DOI:** 10.1002/jha2.136

**Published:** 2021-03-12

**Authors:** Mona Hassanein, Riad El Fakih, Walid Rasheed, Syed Ahmed, Marwan Shaheen, Naeem Chaudhri, Fahad Alsharif, Shad Ahmed, Amr Hanbali, Alfadel AlShaibani, Feras Alfraih, Saud Alhayli, Tusneem Elhassan, Ali Alahmari, Hazzaa Alzahrani, Fahad Almohareb, Mahmoud Aljurf, Shahrukh Hashmi

**Affiliations:** ^1^ King Faisal Specialist Hospital and Research Center Riyadh Saudi Arabia; ^2^ Department of Hematology Bone Marrow Transplant, King's College Hospital NHS Foundation Trust London UK; ^3^ Department of Medicine Mayo Clinic Rochester Minnesota

## Abstract

Secondary acute myeloid leukemia (sAML) includes AML as a complication of an antecedent hematological disorder or a therapy‐related AML. Large registry‐based data identified sAML as an independent poor‐outcome type of AML post allogeneic hematopoietic cell transplantation (allo‐HCT). In our study, we tried to define factors affecting outcomes of sAML post allo‐HCT, and identify patients with sAML who may truly benefit from allo‐HCT. We retrospectively analyzed the data of 64 patients aged (14‐61 years) with sAML who received allo‐HCT between September 2010 and February 2018 at our institute. Most of the patients were transplanted from matched related donors (MRD; 54, 84.4%). Our results showed that poor‐risk cytogenetics were identified in 31 patients (48.4%), and their presence was an indicator of poor overall survival (OS) and disease‐free survival (DFS; *P*‐value = .009, and .004, respectively). The cumulative incidence of chronic graft‐versus‐host disease (cGVHD) was significantly lower in sAML patients with poor‐risk cytogenetics (*P*‐value = .003) resulting in a high risk of death without cGVHD in this group of patients (*P*‐value = .02). Besides, GVHD relapse‐free survival (GRFS) analysis showed that most of our studied patients experienced either relapse or debilitating grade II‐IV cGVHD in the first 2 years post allo‐HCT. We conclude that sAML patients with poor‐risk cytogenetics have a significantly lower DFS post allo‐HCT with a high risk of death without active cGVHD.

## 1 INTRODUCTION

Secondary acute myeloid leukemia (sAML) is a heterogeneous group with poor outcomes compared with de novo AML [1]. It includes AML that develops in patients with a previous hematological disorder, such as myelodysplastic syndrome or chronic myeloproliferative disorder, in addition to AML with a background of dysplasia and therapy‐related AML [2]. The current World Health Organization AML classification defines AML‐myelodysplasia‐related changes and therapy‐related myeloid neoplasms as distinct subcategories [3]. The emergence of AML on top of an antecedent hematological disorder occurs over time, and is believed to be a stochastic process involving random genetic events [4]. Therapy‐related AML (t‐AML) is a clinical syndrome occurring after exposure to cytotoxic and/or radiation therapy [5]. It is believed that sAML constitutes a significant percentage of all AML cases, though it may be underreported due to undiagnosed antecedent hematological disorder and exclusion from clinical trials [6]. Additionally, patients with sAML tend to have dismal outcomes secondary to older age at diagnosis, multiple preceding treatment lines, and poor response to standard intensive chemotherapy among other causes [7].

Allogeneic hematopoietic cell transplantation (allo‐HCT) is the only potentially curative treatment approach for patients with sAML, but many patients are either ineligible or have no appropriate donor[8]. In de novo AML, cytogenetics and molecular markers play an important role in the selection of patients for allo‐HCT [9]. While, in the setting of allo‐HCT in sAML, an early small retrospective study reported no significant effect of patient karyotype on the outcome of sAML when compared to de novo AML post transplant in first complete remission [10]. Till, a later European Society for Blood and Bone Marrow Transplantation (EBMT) registry‐based retrospective study including 11 439 patients with de novo and 1325 with sAML confirmed the Inferior outcome of allo‐HCT for sAML in first complete remission as compared to de novo AML with a statistically significant effect of cytogenetic risk group in a matched‐pair analysis [11]. Furthermore, comorbidities of the patient and transplantation‐associated risk factors may also affect the outcomes [12]. In this study, we analyzed the data of sAML patients who received allo‐HCT at our institute, trying to evaluate the risk factors affecting outcome.

## 2 METHODS

### 2.1 Patients

We reviewed our institutional AML‐transplant database looking for all sAML patients who received their first allo‐HCT at King Faisal Specialist Hospital and Research Center (KFSHRC) between 2010 and 2018. We found a total of 64 patients aged (14‐61 years) with a diagnosis of sAML at time of transplant. We excluded patients with a diagnosis of therapy‐related MDS or patients who had myelodysplasia on top of an antecedent hematological malignancy. The study was approved by our institutional review board (IRB). Cytogenetics at KFSHRC is performed via G‐banding of the metaphases, and was assessed from the laboratory data.

### 2.2 Definitions of outcomes

The primary objective was to study the posttransplant outcomes of these patients in terms of overall survival (OS), disease‐free survival (DFS), acute graft‐versus‐host disease (aGVHD), chronic GVHD (cGVHD), and GVHD relapse‐free survival (GRFS). We studied the correlation of these outcomes with different factors, including disease type (AML/MDS, t‐AML, or AML/MPN), disease status at transplant (CR vs active disease), time from diagnosis to transplant (<6 months vs ≥6 months), transplant type (matched related donors [MRD] vs Haplo or matched unrelated donors [MUD]), and the expression of poor‐risk cytogenetics or monosomy 7.

All outcomes were measured from the time of transplant. DFS was defined as the time until disease relapse or death from any cause. OS was defined as the time until death from any cause. Relapse was defined as the recurrence of the disease. Acute and chronic GVHD were defined according to the standard criteria [13, 14].

The poor‐risk cytogenetics were defined according to 2017 ELN risk stratification [15].

### 2.3 Statistical analysis

Patient characteristics were summarized using frequencies for categorical variables and medians, with ranges for continuous variables. Probabilities of OS and DFS were summarized using Kaplan‐Meier estimator, with variance estimated using Greenwood's formula. Survival curves were compared using the log‐rank test. Probabilities of aGVHD, cGVHD, and relapse were calculated using cumulative incidence function taking into consideration death without GVHD and NRM as competing risks.

Multivariate analysis was utilized to study the impact of proposed risk factors on transplant outcomes such as death, relapse, aGVHD, and cGVHD. Proportionally assumption was tested using time‐dependent covariates. Variables that violate the proportionality assumption were included in the models as time‐dependent covariates. Models were built using forward selection model technique. Covariates with *P*‐value < .05 will be considered significant in the final model. Statistical analysis was carried out using IBM SPSS 20 and R studio.

## 3 RESULTS

More than half of the patients had a diagnosis of AML/MDS (57.8%), 18 patients had t‐AML (28.1%), and the rest of the patients had AML on top of MPN (14.1%). Only two of our studied patients (3.2%) expressed favorable cytogenetics pretransplant and 31 patients (48.4%) had intermediate‐risk cytogenetics. Poor‐risk cytogenetics were identified in 31 patients (48.4%), and monosomy 7 was expressed in 14 of them (21.9%). The stem cells were collected from MRD for 54 patients (84.4%) and from MUD for only four patients (6.2%). The remaining six patients (9.4%) received grafts from Haplo donors. Myeloablative conditioning (Bu/Cy or Cy/TBI for MRD, the same with the addition of two doses of ATG 2.5 mg/kg for MUD, and Bu/Flu/thiotepa with PT‐Cy ATG 1 mg/kg × 2 for Haplo) was used for patients in morphological complete remission at the time of transplant (85.9%) and FLAV‐RIC conditioning (fludarabine/Ara‐C/VP‐16 cytoreduction from D‐13, followed by Bu12/Flu/ATG) was used in patients with active disease (14.1%). All patients received calcineurin inhibitor‐based GVHD prophylaxis with posttransplant cyclophosphamide in the case of Haplo stem cell transplant. The patient's characteristics are shown in Table 1.

**TABLE 1:** Patients characteristics

sAML	Number (total = 64)	%
*Disease type*		
AML/MDS	37	57.8
t‐AML	18	28.1
AML/MPN	9	14.1
*Transplant type*		
MSD	54	84.4
MUD	4	6.2
Haplo	6	9.4
*Time from diagnosis to transplant*		
<6 months	51	79.6
≥6 months	13	20.4
*Disease status at transplant*		
CR	55	85.9
Active disease	9	14.1
*Poor‐risk cytogenetics*		
Yes	31	48.4
No	33	51.6
*Monosomy 7*		
Yes	14	21.9
No	50	78.1

**FIGURE 1:**
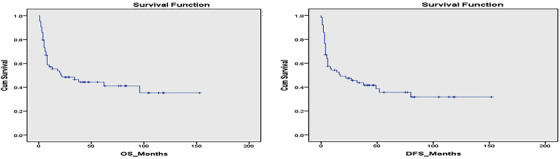
OS and DFS of the studied patients

**FIGURE 2:**
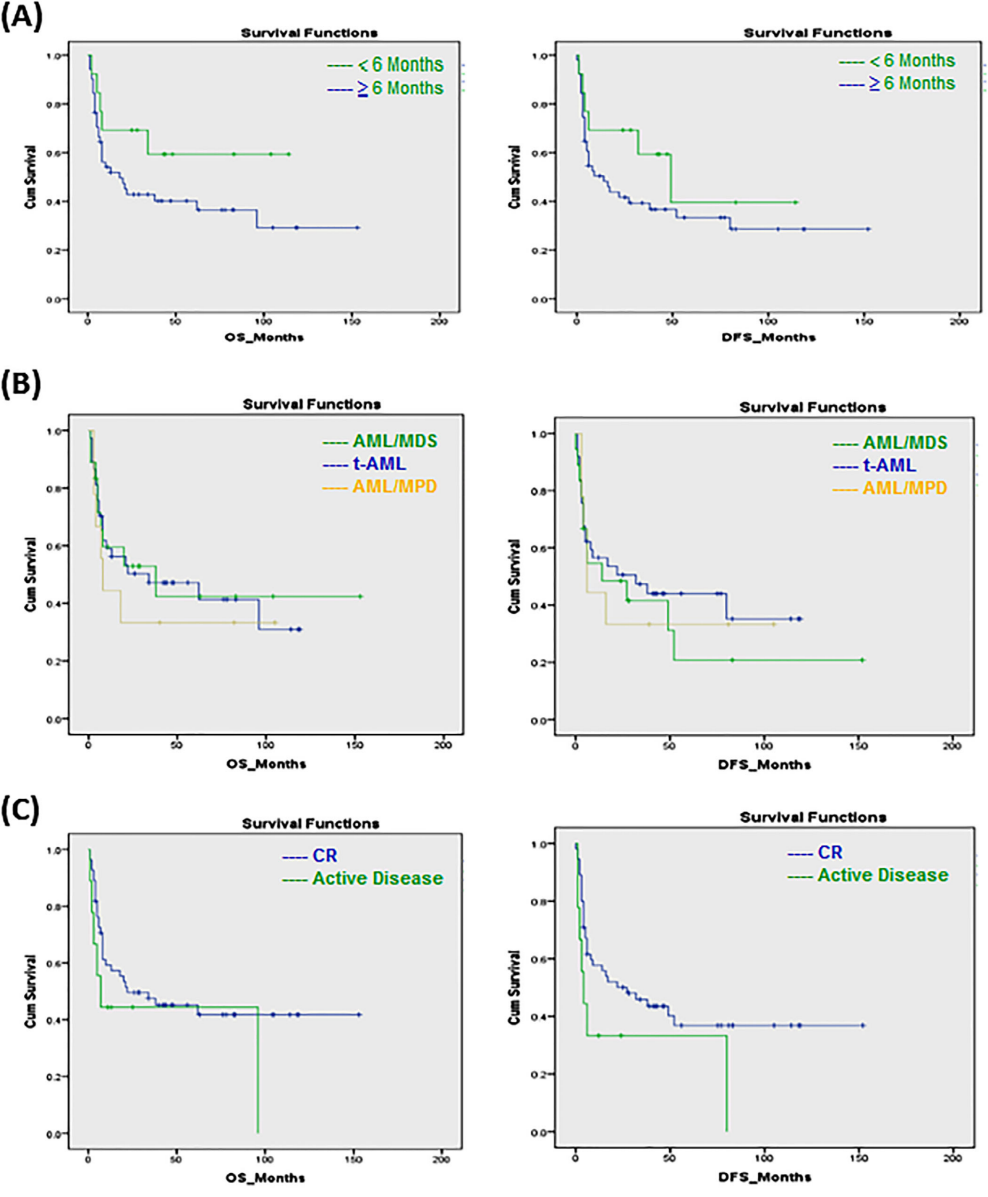
The effect of time from diagnosis to transplant, disease subcategory and disease status on OS and DFS

**FIGURE 3:**
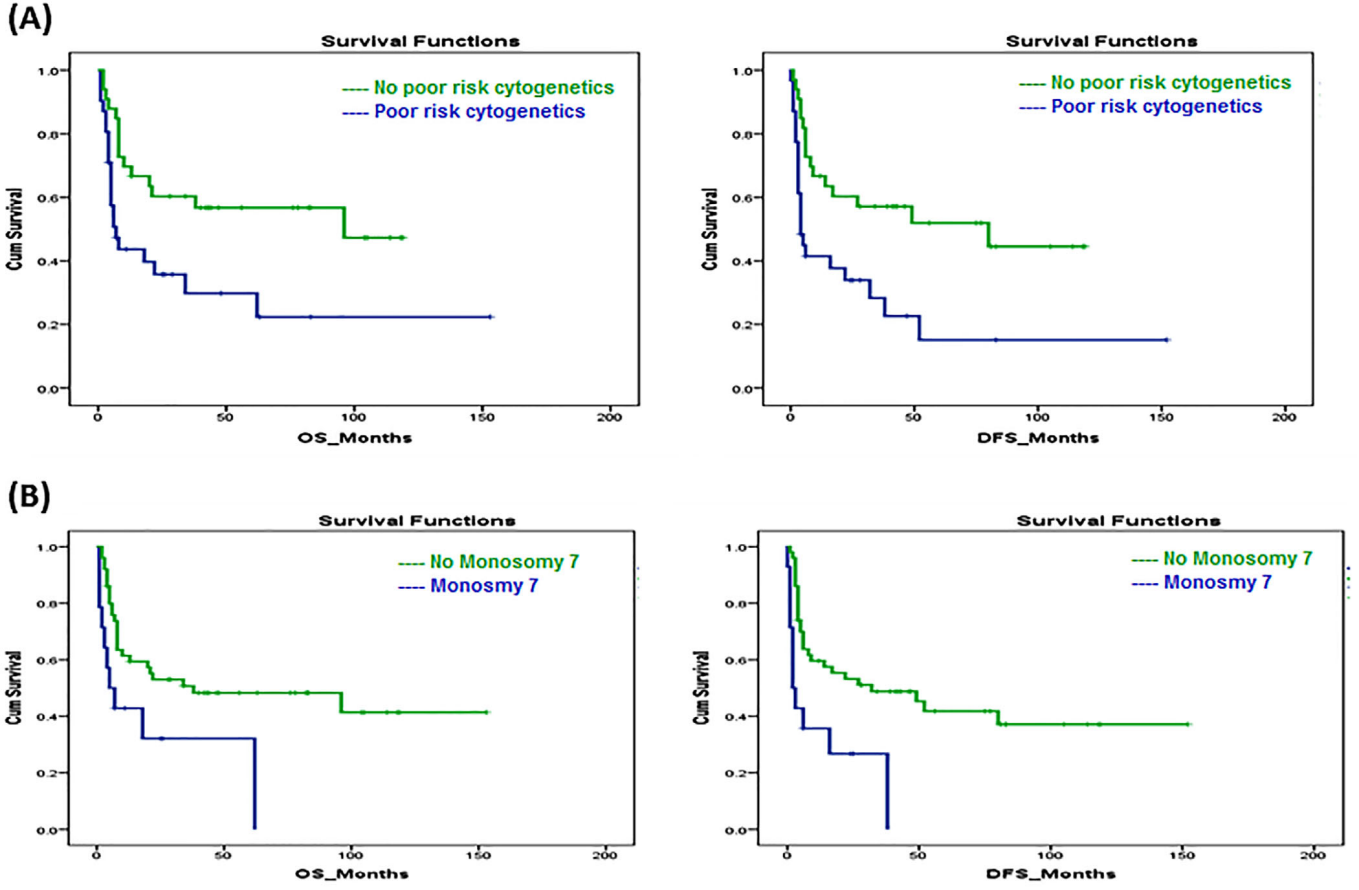
The effect of poor risk cytogenetics and monosomy 7 on OS and DFS

**FIGURE 4:**
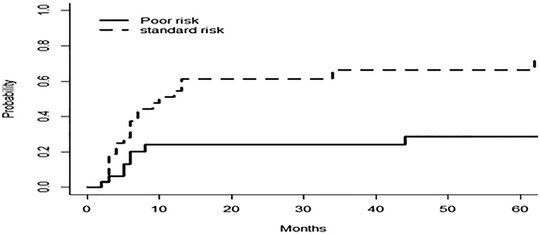
The relation between poor risk cytogenetics and the cumulative risk of cGVHD

**FIGURE 5:**
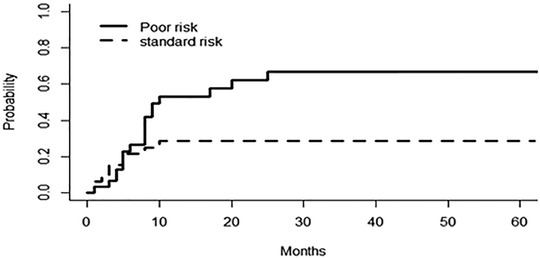
The risk of death in the first 2 years post transplant in absence of cGVHD

**FIGURE 6:**
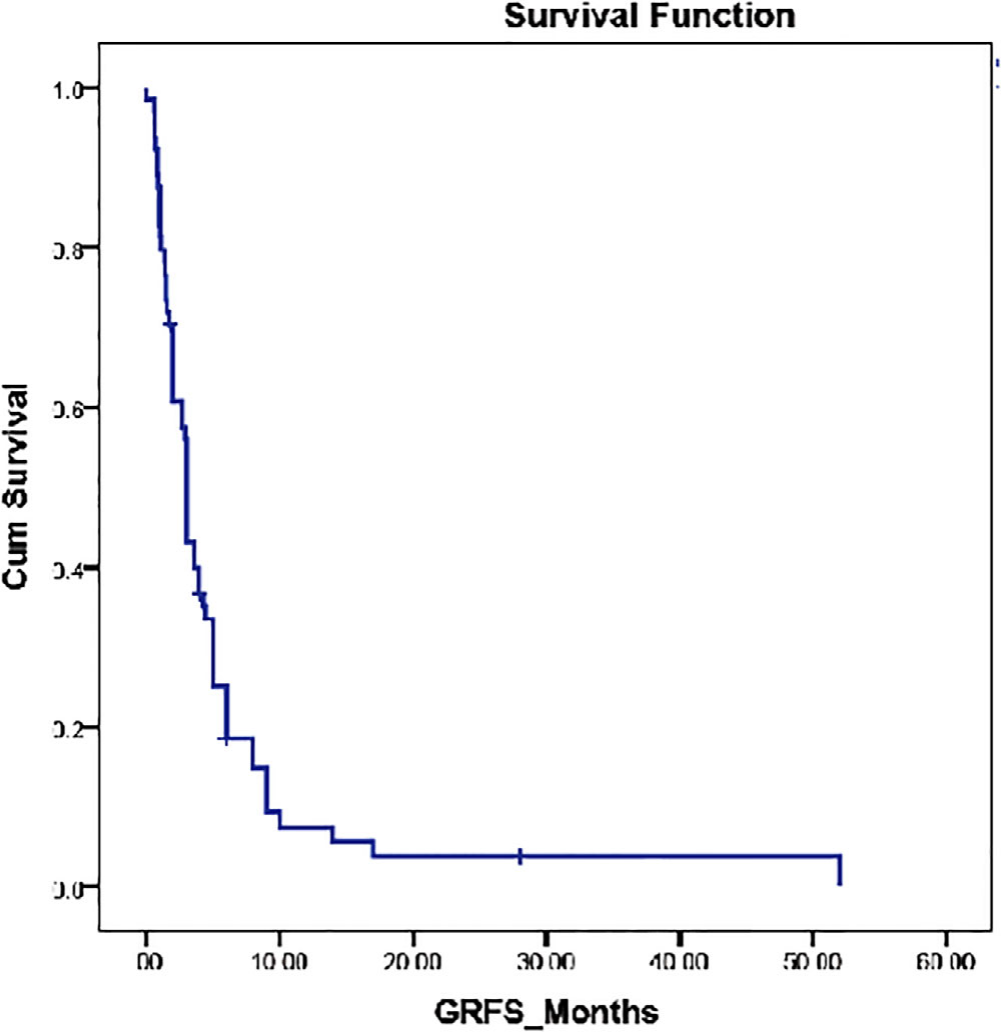
GRFS of the studied patients

The median OS for the studied patients was 22 months, and the median DFS was 17 months at a 12‐year follow up (Figure 1). Our results showed that disease subcategory, time from diagnosis to transplant, and disease status at transplant did not significantly affect the median OS (*P*‐value = .78, .14, and .21, respectively), or the DFS (*P*‐value = .79, .21, and .08, respectively) (Figure 2). However, poor‐risk cytogenetics at diagnosis and the presence of monosomy 7, as a sole aberration or combined with other chromosomal abnormalities, significantly affected OS (*P*‐value = .09 and .015, respectively), and DFS (*P*‐value = .04 and .02, respectively) (Figure 3). The cumulative incidence of aGVHD, cGVHD, or relapse concerning transplant type, disease type, time from diagnosis to transplant, disease status at transplant, the presence of poor‐risk cytogenetics, or monosomy 7 is shown in Table 2. Our results demonstrated that patients with poor‐risk cytogenetics have a significantly lower cumulative risk of cGVHD (*P*‐value = .003; Figure 4), with a high risk of death in the first 2 years post transplant in the absence of cGVHD (Figure 5). GVHD was not used as a competing risk in the statistical analysis. Besides, our studied patients experienced either relapse or grade II‐IV GVHD in the first 2 years post transplant (Figure 6), indicating lack of benefit from the GVL effect of cellular therapy in sAML with poor‐risk cytogenetics.

**TABLE 2:** Transplant outcomes in relation to patient characteristics

	CI of aGVHD	*P*‐value	CI of cGVHD	*P*‐value	CI of relapse	*P*‐value
*Transplant type*		*.29*		*.01*		*.2*
MRD	0.228		0.55		0.4597	
Alternate donor	0		0		0.212	
*Disease type*		*.1*		*.5*		*.3*
AML/MDS	0.2037		0.53		0.353	
AML/MPN	0.062		0.419		0.517	
t‐AML	0.37		NA		0.111	
*Time from Dx to TxP*		*.9*		*.4*		*.9*
˂6 months	0.166		0.505		0.406	
≥6 months	0.277		0.345		0.45	
*Disease status at TxP*		*.4*		*.1*		*.15*
CR	0.201		0.518		0.455	
Active disease	0.111		0.3		0.222	
*Poor‐risk cytogenetics*		*.4*		*.003*		*.4*
Yes	0.202		0.286		0.3218	
No	0.222		0.664		0.563	
*Monosomy 7*		*.27*		*.01*		*.5*
Yes	0.142		0.17		0.297	
No	0.206		0.582		0.467	

## 4 DISCUSSION

Allogeneic transplantation continues to be the only potentially curative treatment choice for patients with sAML. However, the outcomes are not as good as the allo‐HCT outcomes for de novo AML [16, 17]. Herein, we studied the factors that may affect the outcome of patients with sAML after allogeneic transplant. We identified 64 sAML patients who received an allo‐HCT between September 2010 and February 2018, with an age range from14 to 61 years. The large systemic analysis of EBMT reported that older age was associated with inferior survival outcome in a multivariate analysis, and the same with active disease at the time of transplant, and prior other malignant hematological disorder especially lymphoma [1]. Our results failed to show significant differences related to similar risk factors like disease status at time of transplant, disease subcategory, as well as time from diagnosis to transplant either less or more than 6 months. We contribute this failure to a relatively small number of patients included in our study.

However, the effect of cytogenetics and molecular changes on the outcome of sAML treated with allo‐SCT was easily detected in our study as is consistent with multiple other studies [1,8,^18^, ^19^, ^20^]. Poor‐risk cytogenetics were detected in around 50% of our patient population who had a significantly lower OS and DFS, which is comparable to the EBMT registry study data [1]. Besides, monosomy 7 was expressed in more than 20% of our patients, and its presence either alone or combined with other chromosomal abnormalities significantly affected OS and DFS [21].

Similarly, the study by Della Porta et al confirmed the independent clinical significance of mutation screening of ASXL1, RUNX1, and TP53 genes in predicting survival after allogeneic HSCT for patients with MDS and MDS/AML [18]. Furthermore, the outcome prediction model of OS proposed by Heuser and colleagues for MDS and sAML/MDS after allo‐HCT incorporated nine variables, five of these are disease‐related genetic changes including mutated NRAS, U2AF1, IDH2, TP53, and/or a complex karyotype [8]. In our study, at least half of the sAML patients experienced relapse and/or did not survive for 2 years with a median DFS and OS of 17 and 22 months, respectively. This goes along with results of the large study by the acute leukemia working party of the EBMT that reported a 2‐year cumulative incidence of LFS of 38.8% and a 2‐year OS of only 44.5% [1]. However, a small retrospective study showed that allo‐SCT can result in comparable survival outcomes in patients with sAML as seen in patients with de novo AML even after a propensity score‐matched analysis [10]. In our study, we noted that patients with poor‐risk cytogenetics had a high risk of death in the first 2 years post transplant in absence of cGVHD, and the GRFS at 2 years for all patients was less than 10% indicating that most of our studied patients experienced either relapse or grade II‐IV cGVHD in the first 2 years post transplant. Thus, we conclude that sAML patients with poor‐risk cytogenetics do not benefit from GVL effect of allo‐HCT.

Similar to our study results, adverse cytogenetics were associated with inferior GRFS in the largest registry study to date for patients with sAML undergoing transplantation [1]. The limitations of our study include the small number of patients and the lack of subclassification to treated sAML who received at least one therapy for antecedent hematological disorder and proved to have poorer outcomes with low response rates, high early mortality, and higher risk of early disease relapse compared to sAML with untreated AHD [22]. In addition, the lack of use of next‐generation sequencing, which led to major changes in the WHO classification of AML and was proved to have great implications on prognosis and treatment decisions [23].

In summary, our results showed that the expression of poor‐risk cytogenetics characteristically monosomy 7 at diagnosis of sAML is a strong indicator of poor outcome post allogeneic‐HCT. Besides, sAML patients with poor‐risk cytogenetics had a significantly low incidence of cGVHD with a high risk of death in the first 2 years post allo‐HCT, suggesting a potential lack of benefit from GVL effect of cellular therapy in this group of patients. Further prospective studies with the use of advanced diagnostic and follow‐up tools like next‐generation sequencing (NGS) are needed to accurately classify sAML patients according to outcome post allo‐HCT. Lastly, trials evaluating novel agents for the treatment of sAML patients specifically with poor‐risk cytogenetics are urgently needed.

## CONFLICT OF INTEREST

The authors declare that there is no conflict of interest.

## AUTHOR CONTRIBUTIONS

Mona Hassanein and Riad El Fakih wrote the first draft of the manuscript. All the authors contributed substantially to the conception, acquisition, analysis, and interpretation of the data for the work.
